# How uncertain are you? Disentangling expected and unexpected uncertainty in pupil-linked brain arousal during reversal learning

**DOI:** 10.3758/s13415-023-01072-w

**Published:** 2023-02-23

**Authors:** P. Pajkossy, G. Gesztesi, M. Racsmány

**Affiliations:** 1 Department of Cognitive Science, Budapest University of Technology and Economics, Műegyetem rkp 3, Budapest, 1111 Hungary; 2 Institute of Cognitive Neuroscience and Psychology, Research Centre for Natural Sciences, Budapest, Hungary; 3 Institute of Psychology, University of Szeged, Szeged, Hungary; 4 Center for Cognitive Medicine, University of Szeged, Szeged, Hungary

**Keywords:** Expected uncertainty, Unexpected uncertainty, Pupil size, Arousal, Probabilistic reversal learning, Decision-making

## Abstract

During decision making, we are continuously faced with two sources of uncertainty regarding the links between stimuli, our actions, and outcomes. On the one hand, our expectations are often probabilistic, that is, stimuli or actions yield the expected outcome only with a certain probability (expected uncertainty). On the other hand, expectations might become invalid due to sudden, unexpected changes in the environment (unexpected uncertainty). Several lines of research show that pupil-linked brain arousal is a sensitive indirect measure of brain mechanisms underlying uncertainty computations. Thus, we investigated whether it is involved in disentangling these two forms of uncertainty. To this aim, we measured pupil size during a probabilistic reversal learning task. In this task, participants had to figure out which of two response options led to reward with higher probability, whereby sometimes the identity of the more advantageous response option was switched. Expected uncertainty was manipulated by varying the reward probability of the advantageous choice option, whereas the level of unexpected uncertainty was assessed by using a Bayesian computational model estimating change probability and resulting uncertainty. We found that both aspects of unexpected uncertainty influenced pupil responses, confirming that pupil-linked brain arousal is involved in model updating after unexpected changes in the environment. Furthermore, high level of expected uncertainty impeded the detection of sudden changes in the environment, both on physiological and behavioral level. These results emphasize the role of pupil-linked brain arousal and underlying neural structures in handling situations in which the previously established contingencies are no longer valid.

Our decisions are shaped by previous experiences – we tend to choose options associated with positive outcomes in the past (e.g. we will favor restaurants which featured our favorite dish in the past). Importantly, however, the link between choices and rewards is often probabilistic: sometimes an action is associated with reward—sometimes it is not (e.g., when repeatedly visiting the same restaurant, our favorite dish is not always on the menu). Thus, adaptive decision making requires estimating the uncertainty inherent in the links between choices and outcomes. This uncertainty might originate from two separable sources.

On the one hand, based on prior experiences we might establish stable beliefs about the probability with which a stimulus is associated with reward (e.g., I know that in my favorite restaurant, my favorite dish is quite often, but not always on the menu). Uncertainty inherent in such probabilistic stimulus-reward contingencies (SRCs) is often labelled expected uncertainty (Bland & Schaefer, 2012; Soltani & Izquierdo, 2019; Yu & Dayan, 2002, 2005).

On the other hand, sometimes there are drastic and persistent changes in the menvironment (e.g., there is a new chef in my favorite restaurant, and the menu has changed completely). Such environmental change triggers unexpected uncertainty (Bland & Schaefer, 2012; Soltani & Izquierdo, 2019; Yu & Dayan, 2002, 2005), which is uncertainty regarding the validity of the established reward probabilities (e.g., can I still get my favorite food in my favorite restaurant?). Situations characterized by unexpected uncertainty can be resolved by updating our beliefs about the world to reflect the changed SRCs and adaptively guide future decisions (e.g., I don’t expect it anymore to get my favorite dish in my favorite restaurant, and so might choose another restaurant). Importantly, this updating requires that negative feedbacks and reward omissions are attributed to changes in SRCs (unexpected uncertainty) and not to random noise inherent in the probabilistic nature of SRCs (expected uncertainty).

This differentiation between expected and unexpected uncertainty is vital: whereas random fluctuations inherent in established SRCs (expected uncertainty) do not require adaptation, fundamental changes in SRCs (unexpected uncertainty) should lead to the updating and adjusting of SRCs. Coping with unexpected events is accompanied by a general increase in brain arousal, the general excitatory level of the brain (Jones, 2003; Pfaff, 2006; Sokolov, 1963). Through a coordinated activation of several subcortical nuclei, this brain arousal response delivers different neurotransmitters to cortical processing sites (e.g., noradrenaline: Aston-Jones & Cohen, 2005; Sara, 2009; or acetylcholine: Baxter & Chiba, 1999; Rasmusson, 2000) and so modulates cortical processing in a global fashion to facilitate the processing of unexpected uncertainty.

One easily observable indirect measure of this brain arousal is pupil size: increase in pupil diameter can be observed during mental phenomena usually associated with brain arousal (e.g., mental effort and emotional processing: Beatty, 1982; Bradley et al., 2008; Oliva & Anikin, 2018; van der Wel & van Steenbergen, 2018). Furthermore, changes in pupil diameter have been linked to activations of both the noradrenergic and the cholinergic systems (Aston-Jones & Cohen, 2005; Reimer et al., 2016). Accordingly, several studies have found that pupil-linked arousal might signal uncertainty computations in the brain, for example, during perceptual decision making (de Gee et al., 2017; Lempert et al., 2015; Urai et al., 2017) or during the formation of reward expectations (Lavín et al., 2014; Preuschoff et al., 2011; Satterthwaite et al., 2007). Specifically, pupil-linked brain arousal was associated with two important aspects of unexpected uncertainty: on the one hand, pupil size changes were associated with surprise and violation of expectations (De Berker et al., 2016; Lavín et al., 2014; O’Reilly et al., 2013; Preuschoff et al., 2011; Van Slooten et al., 2017; Zénon, 2019), whereas on the other hand, pupil responses were also sensitive to uncertainty resulting from the lack of established SRCs or volatile environment (Muller et al., 2019; Pajkossy et al., 2017, 2018; Vincent et al., 2019). Furthermore, the level of expected uncertainty was also shown to affect baseline pupil size (De Berker et al., 2016) and evoked pupil responses (Nassar et al., 2012).

Our study was designed to complement the above studies in two important ways: First, we investigated the interplay of the afore-mentioned two aspects of unexpected uncertainty in affecting pupil-linked brain arousal: (1) detection of change in SRCs versus (2) uncertainty accompanying the invalidity of previously established SRCs. Interestingly, different conceptualizations of unexpected uncertainty emphasize these two aspects in a somewhat contrasting way: the computational approach put forward by the influential approach of Yu and Dayan (2002, 2005) operationalized unexpected uncertainty as the probability of change in the valid SRCs, whereas for example Soltani and Izquierdo (2019), p. 636) defined it as “*uncertainty* due to subjective perceived changes in reward probabilities” (emphasis added). As summarized in the previous section, both unexpected change and associated uncertainty was linked to pupil-linked brain arousal, and there also are studies where the contributions of both aspects were investigated. For example, Nassar et al. (2012) and Krishnamurthy et al. (2017) showed that evoked pupil dilation is larger for those stimuli, which suggest that the generative process behind the observations has changed. In contrast, uncertainty associated with the features of the underlying generative process was related to baseline pupil size. In a similar approach, Filipowicz et al. (2020) showed that belief uncertainty regarding the source of reward is related to baseline pupil size, whereas surprising stimuli contradicting these beliefs trigger larger evoked pupil dilation.

Importantly, however, in these studies, the two related aspects of unexpected uncertainty were assessed at a different time point: belief uncertainty was calculated before the outcome was presented, whereas the estimate for unexpected change reflected the effect of the outcome. Because of this, these studies could not assess the question whether pupil size changes associated with an outcome reflect change signalized by the new outcome or the uncertainty generated by this change. Furthermore, the dissociation of these two factors is complicated by the fact that change probability and belief uncertainty are positively correlated: when the probability of a change regarding a valid SRC increases, then uncertainty regarding the valid SRC will also increase, because new information is required before certainty regarding the new SRC can be reached. This positive correlation, however, can be avoided, if the SRCs consist of only a limited number of options (e.g., reward is associated with one of two possible sources). In such cases, high probability of change will be associated with low levels of uncertainty regarding the valid SRCs (i.e., if a reward is associated with one of two possible sources then a high probability of change in SRCs means that not the one but the other option will be rewarded). Paradigmatic case for such a scenario is reversal learning, when participants have to figure out which of two response options is associated with reward (or punishment), whereby the rewarded (or punished) response option periodically changes, which is called a reversal. Theoretically, in the case of reversal learning, the link between change probability and uncertainty regarding the valid SRCs resembles an inverted U-shape: uncertainty is low, when change probability (i.e., the probability of reversal) is either low or high. In the former case, the previously rewarded response option is still linked to reward, whereas in the latter case, the other response option becomes the source of reward. In contrast, uncertainty is high, when change probability is at medium levels (i.e., around 0.5). In such cases, there is large uncertainty regarding the rewarded response option. Thus, to further explore how unexpected uncertainty is associated with pupil-linked arousal, we investigated how change probability and uncertainty regarding the SRCs is linked to pupil responses in a reversal learning paradigm. In the case of this task, the link between these two aspects of unexpected uncertainty resembles an inverted U-shape, which enables us to better dissociate its effects.

The second goal of our research was to investigate how the level of expected uncertainty affects pupil-linked brain arousal associated with uncertainty computations. As described previously, the level of expected uncertainty in an environment influences whether unexpected outcomes are attributed to noise inherent in that environment or to fundamental changes. Because of this, the level of expected uncertainty might influence how the pupil-linked arousal system reacts to unexpected uncertainty. In a previous study investigating the link between expected uncertainty and pupil responses, De Berker et al. (2016) instructed participants to choose between two response options from which one option was paired with punishment (electric shock); the SRC between two choice options and punishment was changed gradually from 50%-50% (maximal uncertainty regarding the location of punishment) to 90%-10% (small uncertainty regarding the location of the punishment). Thus, the authors varied the amount of uncertainty inherent in the SRCs (i.e., expected uncertainty) and found larger baseline pupil size accompanying larger uncertainty inherent in the SRCs. Importantly, however, in this study, the level of expected uncertainty changed periodically during the task, and so expected uncertainty was not a stable characteristics of the environment. Furthermore, the interactions between different forms of uncertainty were not investigated, so the interplay between pupil responses, expected, and unexpected uncertainty could not be revealed. In contrast, in the above mentioned study of Nassar et al. (2012), this specific question also was investigated, and the authors found that more noise inherent in the generative process responsible for the observations (i.e., expected uncertainty) predicted neither evoked nor baseline pupil size. Nevertheless, lower level of noise was associated with an increased sensitivity of feedback-evoked pupil responses to prediction errors; the authors suggested that by low levels of noise, prediction errors were attributed to a change in the underlying generative process (and not to a noise environment), and this drove the enhanced pupil response. In our study, we aimed to investigate whether this observed pattern can be also found in a reversal learning paradigm with binary response options.

To investigate the above issues, we conducted two experiments using a probabilistic reversal learning task, where different levels of both expected and unexpected uncertainty can be observed (Soltani & Izquierdo, 2019). Importantly, as pointed out by Soltani and Izquierdo, expected uncertainty can be attributed to the probabilistic nature of SRCs, and so its amount can be objectively defined as a characteristic of the environment. In contrast, unexpected uncertainty is a subjectively perceived change in SRCs, which can only be derived from behavior or from computational modelling. Following this distinction, we constructed a design in which we varied the level of expected uncertainty experimentally and estimated the level of unexpected uncertainty by using a Bayesian model of the task. Specifically, we computed two estimates assessing both aspects of unexpected uncertainty: probability of change in SRCs and uncertainty regarding the validity of the SRCs.

In our experiments, participants engaged in a guessing game on a computer, in which a fictional actor repeatedly hid a stone in one of his hands, and participants had to guess the location of the stone. Correct guesses were rewarded, whereas incorrect guesses were punished. Participants were told that this fictional actor always prefers one of its hands, that is, it tends to hide the stone in its preferred hand more frequently (Fig. 1A). Thus, there was always (1) a preferred, advantageous response option associated with high probability of reward and low probability of punishment, and (2) a disadvantageous, nonpreferred response option with low probability of reward and high probability of punishment. We will refer to these two response options as *preferred* and *nonpreferred* response options, respectively (because they are preferred or not preferred by the fictional actor). Through trial-and-error learning, participants could form SRCs guiding their behavior, and so they could figure out which is the preferred response option. Crucially, we introduced sudden changes into the learning environment by occasionally switching the preferred and nonpreferred choice options (i.e., the previously preferred choice option became the nonpreferred choice option). During such reversals, the previously valid SRCs became invalid and had to be updated, which triggered unexpected uncertainty.

**Fig. 1:**
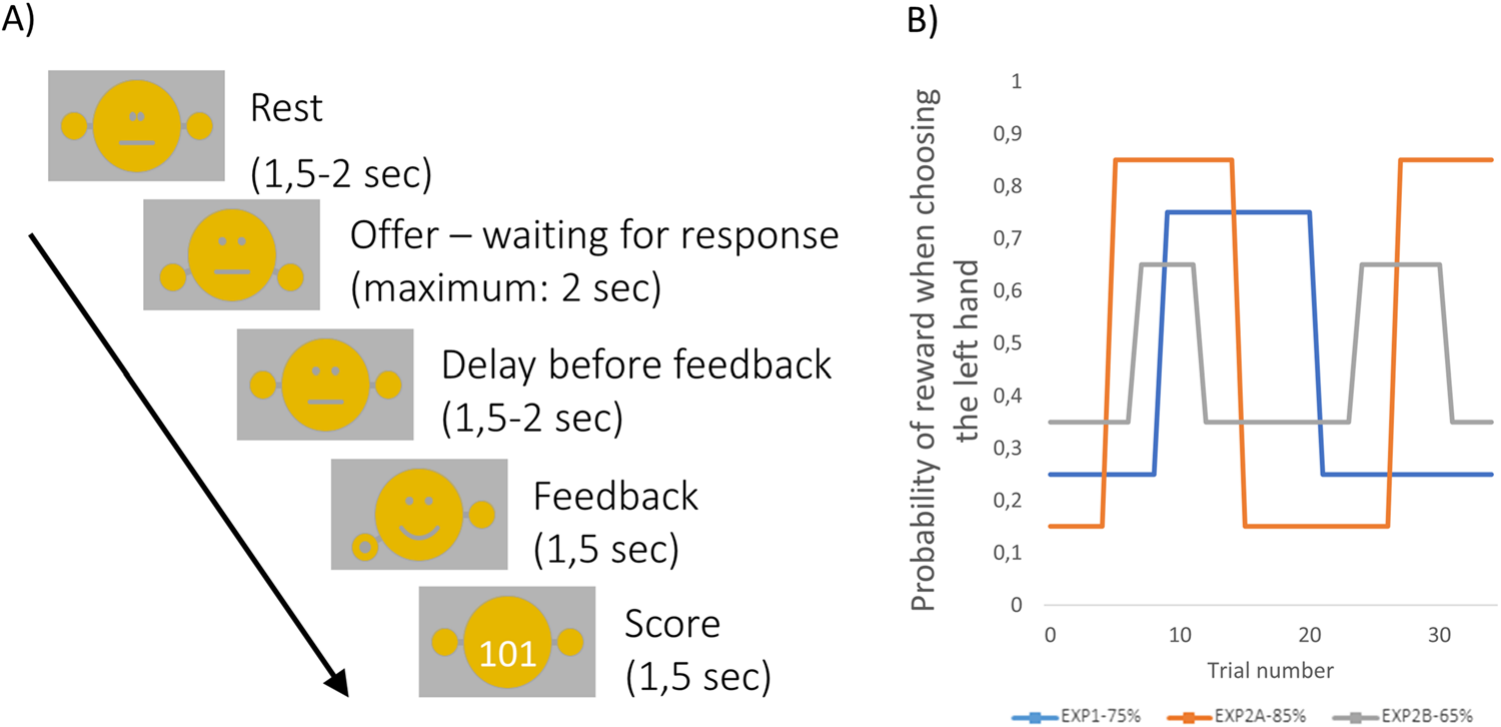
Task structure. **A** Structure of a trial. **B** Stimulus reward contingencies in the different experimental conditions

To estimate change probability and the level of uncertainty related to the validity of SRCs, we defined a Bayesian model of the task. We used a Hidden Markov Model (HMM) to evaluate the level of unexpected uncertainty related to each experimental trial. In this HMM, the observations are the feedbacks received in each trial, and the hidden state is the preferred choice option in that trial. This model estimates for each trial how an ideal Bayesian observer would represent the probability that one or the other response option is the preferred one. On some trials, these probabilities strongly favored one of the response options (e.g., the probability of the left hand to be the preferred response option was 0.95, whereas the probability of the right hand to be the preferred response option was 0.05). In these trials, participants might have a strong belief about the preferred response option, so uncertainty regarding the valid SRC was low. In other trials, the model estimates suggested that the two response options are equally probable (e.g., the probability of both hands to be the preferred response option was around 0.5). Such probabilities might characterize periods when the validity of participants’ beliefs is questioned, and so uncertainty regarding the valid SRC was high. The reciprocal relation of the probabilities corresponding to the two response options can be quantified by the information theoretical measure of entropy (Shannon, 1948), with smaller values corresponding to trials when one of the response options is more probable and larger values corresponding to trials when both response option have a probability around 0.5. Because of this, the entropy associated with the hidden state of the model (i.e., the preferred response option), will be termed *state entropy*, and it will be used to assess uncertainty regarding the valid SRC.

We also estimated the probability of change in the SRCs (i.e., the probability of reversal). Specifically, we calculated the probability that the preferred response option (i.e., the hidden state in the HMM) has been changed since the previous trial. We used the experimental subject’s response in the previous trial as reference. So if the subject selected left in the previous trial, then state change probability is the probability given by our model that the current preferred side is the right. Similarly, if the subject selected right in the previous trial, then state change probability is the probability that the current hidden state is left. This measure represents the probability that the hidden state has changed; thus, we will use the term *state change probability* when using it to assess the probability that the SRCs have changed. Note that this operationalization of unexpected uncertainty is similar to that put forward by Yu and Dayan (2002, 2005).

Importantly, state entropy and state change probability are deterministically linked: state entropy can be calculated as a function of state change probability. This is because the entropy function of the Bernoulli distribution is symmetrical, so it does not matter if we calculate entropy from the probabilities of left and right or the probabilities of changed and unchanged. State entropy is maximal (1) if state change probability is 0.5, and 0, if state change probability is either 0 or 1. Thus, the link between the two variables resembles an inverted U-shape (see Fig. 2B in the *Methods* section), which fits with the theoretical considerations about the relation between change probability and resulting uncertainty in a reversal paradigm (see above), and enables us to dissociate their respective effects.

**Fig. 2:**
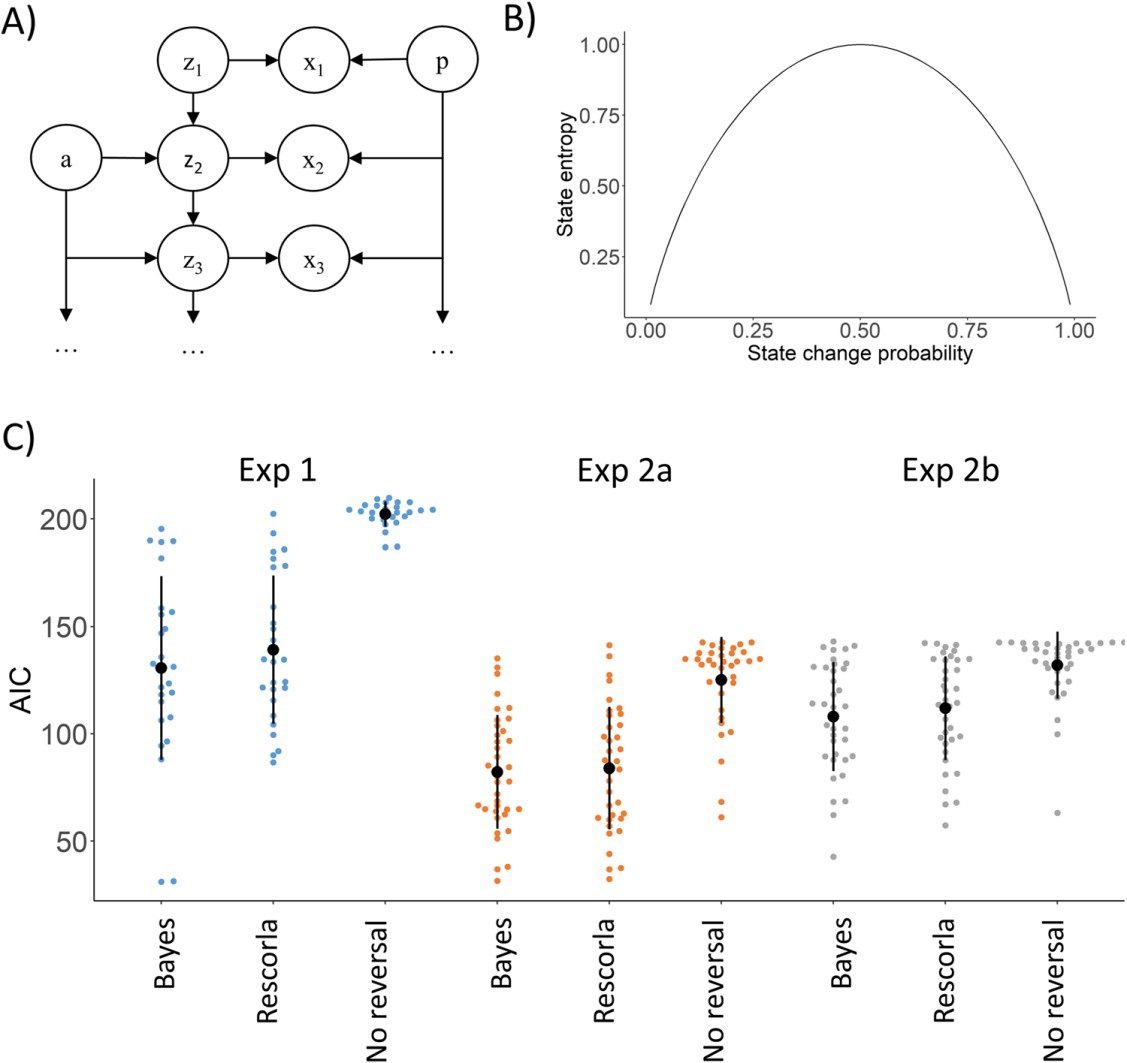
Modelling unexpected uncertainty. **A** Bayesian network of the HMM (Hidden Markov Model) used to evaluate the unexpected uncertainty of the experimental trials. **B** The relationship between state change probability and state entropy. **C** Comparing our Bayesian model with the alternatives using AIC (Akaike Information Criterion). AIC quantifies how well each model describes the participants’ behavior. It is corrected for the number of free parameters

Crucially, we varied the level of expected uncertainty by using different SRCs associated with preferred and non-preferred response options, respectively. In Exp 1, the preferred response option was rewarded with a probability of 0.75 (i.e., the probability of reward in the non-preferred response option was 0.25). In Exp 2, all subjects performed the task with two different SRCs: in one condition (Exp 2a), reward probability was 0.85 for the preferred, and 0.15 for the nonpreferred response option, whereas in the other condition (Exp 2b), reward probability was 0.65 for the preferred, and 0.35 for the nonpreferred response option. Thus, by applying different SRCs in the three *experimental conditions* of the two experiments (Exp 1: 0.75/0.25 vs. Exp 2a: 0.85/0.15 vs. Exp 2b: 0.65/0.35), we varied the magnitude of expected uncertainty and investigated whether it influenced unexpected uncertainty, as measured by state entropy, and pupil-linked brain arousal.

Finally, we were interested in pupil size changes during and after the processing of the feedback, because this is the period where participants are processing information relevant for estimating and adjusting the probabilities associated with the preferred choice option. Unexpected uncertainty is triggered by observations which do not fit the previously established regularities of the environment, so effects related to unexpected uncertainty are bound to the specific observations. Because of this, in contrast to previous studies using a broader scope (De Berker et al., 2016; Filipowicz et al., 2020; Krishnamurthy et al., 2017; Nassar et al., 2012), we restricted our focus to the investigation of evoked changes, and did not examine effects related to baseline pupil size, which are influenced by previous observations.

## Methods

### Participants

Participants were undergraduate and graduate students from different Hungarian universities. They received either partial course credit (Exp 1) or money (Exp 2) for their participation. The research project was approved by the United Ethical Review Committee for Research in Psychology, Hungary. The sample size was N = 26 for Exp 1 (14 females, age range: 19-31 years, M_age_ = 22.57, SD = 2.73) and N = 37 for Exp 2 (25 females, age range: 18-26 years, M_age_ = 22.21, SD = 2.11).

Participants had normal or corrected to normal vision. We asked them to refrain from the consumption of alcohol, caffeine, and nicotine on the day of the experiment. All participants provided informed consent.

### Experimental design and procedure

Participants took part in a probabilistic reversal-learning task. The task was administered on a personal computer, using the OpenSesame graphical experiment builder and presentation software (Mathôt et al., 2012).

Participants were asked to play a game with a small fictional actor depicted as a stick figure on the screen (Fig. 1A). In each trial, the actor hid a stone in one of its hands and the participant had to guess if the stone was in its left or right hand. If the guess was correct then one point was given to the participant, otherwise one point was taken from the participant. The game started from 100 points. The players had to collect as many points as they could.

Subjects had been previously informed that the actor always had a preferred hand, and the stone was hidden more often (but not exclusively) in that hand. They also had been told that sometimes, but not very frequently, the actor changes the preferred hand, and that to collect points, they have to keep track of these changes.

In Exp 1, each participant performed 150 successive trials. In each trial, the stone was hidden with 0.75 probability in the preferred hand and with 0.25 probability in the nonpreferred hand (Fig. 1B). Initially, in the first trial, the preferred hand was selected at random (with 0.5-0.5 probabilities). The hand preference remained unchanged until the participant selected the preferred hand in eight consecutive trials. Then, the preferred and nonpreferred hands were switched. For the switch to happen, it was not important if the guess was rewarded or not, only that it was the preferred direction. If the participant picked the nonpreferred hand, then the count for preferred responses started again from zero.

This behavior necessarily implied that participants possessed strong beliefs about the location of the preferred choice option and so reversal of SRCs caused unexpected uncertainty. Thus, in Exp 1, we aimed to ensure that reversals are associated with strong increase in unexpected uncertainty. Such design with a response criterion, however, also has drawbacks, because it makes reversals and unexpected uncertainty contingent on choice behavior of the participants (Soltani & Izquierdo, 2019). Due to this, in Exp 2 reversals did not depend on the participants’ responses.

Exp 2 consisted of two independent blocks each containing 100 successive trials. We refer to these blocks as Exp 2a and Exp 2b. The players could rest a few minutes between the two blocks. They were informed that the actor’s preferences have been reset. Scores also were restarted in the second blocks. In Exp 2a, the stone was hidden with 0.85 probability in the preferred hand and with 0.15 probability in the nonpreferred hand. In Exp 2b, these probabilities were 0.65 and 0.35, respectively (Fig. 1B). In all other aspects, the two blocks were identical.

Unlike in Exp 1, the switches did not depend on the player’s responses. The preferred hand was switched independently with 0.06 probability after each trial. The presentation of Exp 2a and Exp 2b was conducted by the participants in a counterbalanced order: half of the participants started with Exp 2a, whereas the other half of the participants with Exp 2b.

### Structure of a trial

Each trial started with a fixation phase, during which the fictional character looked straight ahead with his arms at rest. The duration of this fixation phase was between 1.5-2 seconds, the current value drawn in each trial from a uniform distribution. Then, the character showed off his closed hands indicating that the participant could choose. The participant had 2 seconds to pick either the left or the right hand using the arrow keys. Upon selection the character pulled back his hands, indicating that the selection was done. This was followed by a 1.5–2-second-long wait period, drawn from a uniform distribution, so that we could separate the response-related and feedback-related pupillary changes. This step was omitted if the participant did not select during the available time.

Finally, visual feedback was provided. This had two subphases each taking 1.5–1.5 seconds. First, the character opened his hands so that the position of the stone was visible. He was also smiling (if the participant won) or sad (if the participant lost). The face of the character was replaced with a clock if the participant did not respond. During the second subphase of the feedback the participant’s total score was presented. The stimulus outlay for the different trial phases is presented on Fig. 1A.

### Modelling unexpected uncertainty

We used a Hidden Markov Model (HMM) to evaluate the level of unexpected uncertainty related to each experimental trial. The observations are the feedbacks received in each trial and the hidden state is the preferred choice option in that trial. Using this model, we calculated the probability for the two choice options to be the preferred choice option after each successive feedback. The Bayesian network (graphical model) representation of the model is shown in Fig. 2A.

The hidden variable *z*_*t*_ is the preferred direction in trial *t*. In the first trial the directions 0 (left) and 1 (right) have equal probabilities (Eq. 1).

1 $$P\left({z}_1=0\right)=P\left({z}_1=1\right)=0.5$$

The random variable *a* specifies the probability that the preferred direction is changed between a trial and the subsequent one (Eq. 2).

2 $$P\left({z}_{t+1}|{z}_t,a\right)=\left\{\begin{array}{c}1-a,\kern0.5em {z}_{t+1}={z}_t\\ {}a,\kern0.5em {z}_{t+1}=1-{z}_t\end{array}\right.$$

We assumed a beta prior distribution for *a* (Eq. 3).

3 $$a\sim Beta\left({\alpha}_a,{\beta}_a\right)$$

Values *α*_*a*_ and *β*_*a*_ are free parameters of the model.

The observed variable *x*_*t*_ is the correct response in trial *t*. The *p* random variable specifies the probability that the correct response is the same as the preferred direction at any given trial (Eq. 4).

4 $$P\left({x}_t|{z}_t,p\right)=\left\{\begin{array}{c}p,\kern0.5em {x}_t={z}_t\\ {}1-p,\kern0.5em {x}_t=1-{z}_t\end{array}\right.$$

The prior distribution of *p* is assumed to be a beta distribution truncated to the [0.5, 1] interval (Eq. 5). The truncation is made to ensure that *z*_*t*_ is actually the preferred direction, i.e., it is selected with a probability greater than 0.5.

5 $$pdf(p)\propto \left\{\begin{array}{c} pd{f}_{Beta}\left(p;{\alpha}_p,{\beta}_p\right),\kern0.5em 0.5<p\le 1\\ {}0,\kern0.5em otherwise\end{array}\right.$$

The values *α*_*P*_ and *β*_*P*_ are also free parameters of the model.

#### Calculating model probabilities

In theory the hidden state probabilities in a trial can be calculated by summing for all possible ***z***_**1 :** ***t*** **− 1**_ historical values of the hidden variable (Eq. 6).

6 $$P\left({z}_t=z|{x}_{1:t}\right)=\frac{\sum_{z_{1:t-1}}P\left({z}_{1:t-1},{z}_t=z,{x}_{1:t}\right)}{\sum_{z_{1:t}}P\left({z}_{1:t},{x}_{1:t}\right)}$$

In practice, however, this quickly becomes intractable, because the number of terms grows exponentially. To handle the situation, we used the Sequential Monte Carlo (particle filtering) method. A fixed size sample (a fixed number of “particles”) was used to approximate the right side of Eq. 6. Each particle represents a certain *z*_1 : *t* − 1_ hidden state history. Initially, all particles represent the “empty” history, and they have equal probabilities.

For each new trial, we calculated the particle probabilities for both possible hidden state (*z*_*t*_) and observation (*x*_*t*_) values. In the first trial, these are calculated according to Eq. 7, which is derived from Eq. 1 and Eq. 5.

7 $$P\left({z}_1,{x}_1\right)=\left\{\begin{array}{c}\frac{1}{2}\bullet \frac{\alpha_p+{c}_0}{\alpha_p+{\beta}_p},\kern0.5em {x}_1={z}_1\\ {}\frac{1}{2}\bullet \frac{\beta_p-{c}_0}{\alpha_p+{\beta}_p},\kern0.5em {x}_1\ne {z}_1\end{array}\right.$$

The *c*_0_ correction term is necessary, because we assumed a truncated beta distribution for *p*. It can be calculated according to Eq. 8. (The functions Β(*α*, *β*) and Β(*p*; *α*, *β*) are the beta function and the incomplete beta function respectively.)

8 $${c}_0=\frac{0.5^{\alpha_p+{\beta}_p}}{\textrm{B}\left({\alpha}_p,{\beta}_p\right)-\textrm{B}\left(0.5;{\alpha}_p,{\beta}_p\right)}$$

In the subsequent trials, the new particle probabilities can be calculated using the chain rule (Eq. 9).

9 $$P\left({x}_{1:t+1},{z}_{1:t+1}\right)=P\left({x}_{1:t},{z}_{1:t}\right)P\left({z}_{t+1}|{z}_{1:t}\right)P\left({x}_{t+1}|{x}_{1:t},{z}_{1:t+1}\right)$$

The conditional probabilities are calculated according to Eq. 10 and Eq. 11.

10 $$P\left({z}_{t+1}|{z}_{1:t}\right)=\left\{\begin{array}{c}\frac{\beta_a+t-1-{r}_t}{\alpha_a+{\beta}_a+t-1},\kern0.5em {z}_{t+1}={z}_t\\ {}\frac{\alpha_a+{r}_t}{\alpha_a+{\beta}_a+t-1},\kern0.5em {z}_{t+1}\ne {z}_t\end{array}\right.$$

11 $$P\left({x}_{t+1}|{x}_{1:t},{z}_{1:t+1}\right)=\left\{\begin{array}{c}\frac{\alpha_p+{m}_t+{c}_t}{\alpha_p+{\beta}_p+t},\kern0.5em {x}_{t+1}={z}_{t+1}\\ {}\frac{\beta_p+t-{m}_t-{c}_t}{\alpha_p+{\beta}_p+t},\kern0.5em {x}_{t+1}\ne {z}_{t+1}\end{array}\right.$$

Here, *r*_*t*_ denotes the number of reversals until trial *t*, while *m*_*t*_ is the number of trials until *t* where the preferred direction was selected (*x*_*τ*_ = *z*_*τ*_). The *c*_*t*_ correction term also can be calculated recursively using Eq. 8 and Eq. 12.

12 $${c}_{t+1}=\left\{\begin{array}{c}\frac{0.5\left({\alpha}_p+{\beta}_p+t\right){c}_t}{\alpha_p+{m}_t+{c}_t},\kern0.5em {x}_{t+1}={z}_{t+1}\\ {}\frac{0.5\left({\alpha}_p+{\beta}_p+t\right){c}_t}{\beta_p+t-{m}_t-{c}_t},\kern0.5em {x}_{t+1}\ne {z}_{t+1}\end{array}\right.$$

Note that Eq. 6 only contains the ratio of the summed probabilities and the denominators in Eq. 10 and Eq. 11 do not depend on any of the *x*_*i*_ or *z*_*i*_ variables. Thus, we can simplify Eq. 6 with them so they are not needed in the actual calculation. For the same reason, we can renormalize particle probabilities at each step to add up to 1. This way we can avoid underflow in the numerical calculation.

When the feedback of the trial is available we keep only the particle probabilities matching the observation. These are then resampled to keep the number of particles fixed.

#### Using the model to estimate unexpected uncertainty

The main purpose of our model is to estimate the unexpected uncertainty in each trial. Yu and Dayan (2002, 2005) defined unexpected uncertainty as the probability that the SCRs underlying a task have been drastically changed, and the learned contingencies became essentially invalid. In our case, this happens at reversals. To capture the event that a reversal has happened, we need a reference hidden state value representing the preferred side the participant has already learned. The best proxy for this is arguably the actual selection of the subject. We denote it as ***y***_***t***_. So the unexpected uncertainty according to Yu and Dayan (2002, 2005) can be quantified by the state change probability of Eq. 13.

13 $$state\_ change\_{probability}_t=P\left({z}_t\ne {y}_t|{x}_{1:t}\right)$$

If SCRs are represented by continuous values (Yu & Dayan, 2002) or there are presumably many possible SCRs (Yu & Dayan, 2005), then a higher probability that these SCRs have been changed usually means less information about the current SCRs. However, this is not always necessarily the case. In our reversal-learning task for example, always either the left or the right hand is preferred. So a very high state change probability (>0.5) actually means that the subject can be relatively certain that the preferred side is the opposite of the selected one. So the state change probability actually measures unexpected change rather than uncertainty in the sense of lack of information. The least is known about the hidden state, i.e., the uncertainty about it is highest if state change probability is 0.5. Based on our data trials with state change, probability above 0.5 are relatively common.

To measure unexpected uncertainty in the sense of lack of information about the current SCRs (as opposed to unexpected change above), we also calculated the entropy from the hidden state probabilities using the classic formulation of entropy defined by Shannon (1948) (Eq. 14).

14 $$state\_{entropy}_t=H\left({z}_t|{x}_{1:t}\right)=-\sum_zP\left({z}_t=z|{x}_{1:t}\right)\bullet logP\left({z}_t=z|{x}_{1:t}\right)$$

It is important to note that because ***y***_***t***_ is known and the binary entropy function is symmetric it makes no difference if we calculate the entropy from the probabilities that the hidden state is left/right or same as/opposite of ***y***_***t***_. So state entropy can be calculated as a function of state change probability using the binary entropy function. The relationship between state change probability and state entropy is shown in Fig. 2B.

- ***Parameter fitting:*** We used the maximum likelihood method to estimate the free model parameters based on the selections of the subjects. For this, we also needed a choice model. The choice model is used to calculate the probabilities that the subject is selecting the left or the right side based on the probabilities that the stone is in the left or the right hand as predicted by the HMM model. We used the “softmax” rule (Eq. 15) as choice model.

15 $$P\left({y}_t=y|{x}_{1:t-1}\right)=\frac{\mathit{\exp}\left(\beta P\left({x}_t=y|{x}_{1:t-1}\right)\right)}{\sum_x\mathit{\exp}\left(\beta P\left({x}_t=x|{x}_{1:t-1}\right)\right)}$$

Here, *y*_*t*_ is the subject’s actual choice in trial *t*. The parameter *β* is known as the inverse temperature. It controls the level of stochasticity in the choice. The *β* = 0 case means completely random choice while *β* = ∞ is always selecting the side with the higher probability. *β* is treated as an additional free parameter.

Thus in total, our model has five free parameters (Eq. 16). Parameters *α*_*p*_ and *β*_*p*_ specify the subject’s prior expectations about the distribution of *p*, that is the probability of selecting the preferred direction. Parameters *α*_*a*_ and *β*_*a*_ specify the subject’s prior expectations about the distribution of *a*, that is the probability of reversal between two subsequent trials. *β* is the inverse temperature from the choice model.

16 $$\theta =\left({\alpha}_p,{\beta}_p,{\alpha}_a,{\beta}_a,\beta \right)$$

Maximum likelihood parameter estimation is used to determine the parameter values where the probability of the options actually selected by the experimental subject given all the prior observations, and the *θ*^∗^ parameter values is maximal (Eq. 17).

17 $${\theta}^{\ast }=\underset{\theta }{\textrm{argmax}}\prod_tP\left({y}_t|{x}_{1:t-1};\theta \right)=\underset{\theta }{\textrm{argmin}}\left(-\sum_t logP\left({y}_t|{x}_{1:t-1};\theta \right)\right)$$

The estimation is performed separately for each subject. In the case of Exp 2, the parameters are estimated separately for the two blocks (Exp 2a and Exp 2b). The optimization was performed using the Sequential Least Squares Programming (SLSQP) method. We used multiple random starting points to ensure convergence. The best result was kept.

#### Model validation

Although the model corresponds to the task and instructions given to the experimental subjects, it still needs to be validated that the subjects indeed perform this task. In particular, the rate of learning does indeed depend on the amount of unexpected uncertainty. We considered two competing models.

The first is a reinforcement learning model combining the Rescorla-Wagner (Rescorla & Wagner, 1972) update rule (Eq. 18) with the “softmax” choice rule (Eq. 19).

18 $${Q}_{t+1}^y={Q}_t^y+\alpha \left({R}_t-{Q}_t^y\right)$$

The term ${Q}_t^y$is the expected reward for side *y* at trial *t*, *R*_*t*_ is the received reward at *t* and *α* is the learning rate.

19 $$P\left({y}_t=y|{x}_{1:t-1}\right)=\frac{\mathit{\exp}\left(\beta {Q}_t^y\right)}{\sum_x\mathit{\exp}\left(\beta {Q}_t^x\right)}$$

We assumed that ${Q}_1^0={Q}_1^1=0.5$, so the model has two free parameters: the learning rate and the inverse temperature (Eq. 20).

20 $$\theta =\left(\alpha, \beta \right)$$

The second competing model is the limiting case of our Bayesian model, where *α*_*a*_ = 0 and *β*_*a*_ = ∞. This means that *a* = 0 deterministically, so no reversals are possible. We refer to this as no reversal model. In this special case, model probabilities can be calculated analytically according to Eq. 21, so no Monte Carlo simulation is necessary. (For simplicity we assumed a nontruncated beta distribution for *p* in this case.)

21 $$P\left({x}_{t+1}=1|{x}_{1:t}\right)=P\left({x}_t=1|{x}_{1:t-1}\right)+\frac{x_t-P\left({x}_t=1|{x}_{1:t-1}\right)}{\alpha_p+{\beta}_p+t}$$

Note that it is essentially a reinforcement learning update rule with a hyperbolically decreasing learning rate. Additionally, the update only depends on the sum of *α*_*p*_ and *β*_*p*_, so the model actually has only two free parameters (Eq. 22).

22 $$\theta =\left({\alpha}_p+{\beta}_p,\beta \right)$$

Before evaluating which of the models fit best the responses of the experimental subjects, we tested model recovery with simulated data. We used the same tasks and task parameters as in the experiments (Exp 1, Exp 2a, and Exp 2b) to generate simulated data. For each model and experiment, the same number of simulated sessions were generated as the number of real subjects. The distribution of the model parameters also were the same as we obtained by the maximum likelihood parameter fitting of the experimental data. Then, using only the responses and the feedbacks, we fit each model to each of the simulated datasets. Then, we calculated the Akaike Information Criterion (AIC) and Bayes Information Criterion (BIC) values to check if they can be used to recover the model used to generate each dataset.

We found that BIC could not be reliably used for model recovery even with the simulated data given the specific tasks, parameters and sample size. In particular for Exp 2 paired *t*-tests showed no significant difference in the BIC values of fitting the HMM and Rescorla-Wagner models when the dataset was actually generated by the HMM model (Exp 2a: *p* = 0.94, Exp 2b: *p* = 0.59). The simulated Exp 1 experiment was significant (*p* = 0.04).

On the other hand, AIC reliably indicated which model was used to generate a particular dataset. Paired *t*-tests showed significantly lower AIC values when fitting the same model used for the dataset generation as compared to fitting a different competing model (*p* < 0.01 for all cases, but *p* < 0.001 if one of the models is HMM). Therefore, we used AIC for the model validation.

Then, we calculated the AIC values for the maximum likelihood fitting of each model to the experimental data (Fig. 2C). For each experiment, the smallest mean AIC value corresponded to the HMM model. The difference between the mean AIC values of the HMM and the no reversal models were highly significant (*p* < 0.001 for all experiments). The difference between the mean AIC values of the HMM and Rescorla-Wagner models were significant for Exp 1 (*p* = 0.046) and Exp 2b (*p* = 0.012). There was no significant difference between the mean AIC values of the HMM and Rescorla-Wagner models in the case of Exp 2a (*p* = 0.21). The comparison of the AIC values are shown in Fig. 2C.

Overall, the validation result supports our claim that the HMM model better describes the behavior of the experimental subjects than the competing models, which do not consider the differences between the high and low unexpected uncertainty trials.

### Eye tracking

Subjects were seated in a dimly lit room with their head resting on a chin rest 60-cm away from a 23-inch monitor. The stimulus was presented at the center of the screen (approximate viewing angle: 3^0^) using isoluminant colors on a homogenous grey background. We used an SMI RED500 remote eye-tracker system (SensoMotoric Instruments, Teltow, Germany) mounted to the bottom of the monitor, with a sampling rate of 250 Hz. Pupil data from both eyes were recorded.

### Pupil data preprocessing

The pupil data were preprocessed to reduce noise and remove blinks or other artefacts. First, the pupil diameters of the two eyes were averaged. Then, data points during blinks were deleted. We used the eye-tracker’s own event detector software to detect blinks using the default parameters. We treated as blink if the software reported blink for at least one eye.

Data points in each data set, which deviated from the mean of the data series by more than 3 SDs, were deleted to remove high frequency noise. Ten data points (40 msec) before and after such segments also were removed.

The remaining data were resampled at 50 Hz, and missing data points were interpolated using linear interpolation and then smoothed using Savitzky-Golay filter (frame size: 9, polynomial order: 4). The mean percentage of interpolated data were M = 5.52% (range: 1.94-18.81%, SD = 3.61%) in Exp 1, and M = 8.31% (range: 1.75-25.09%, SD = 2.23%) in Exp2. The ratio of interpolated data points exceeded the sample mean by more than 2.5 SD for one participant in Exp 1 and for two participants in Exp 2; thus, they were excluded from pupil-size analyses. Importantly, for analyzes not involving pupil data, no such exclusions were made.

### Statistical analyses

We were interested in pupil size changes associated with the feedback. Thus, relevant data segments were extracted from the data flow, aligned to the feedback. Pupil data were extracted for the period from 1 second preceding the feedback to 6 seconds following the feedback. To quantify pupil size changes relative to pre-feedback baseline, all data segments were baseline corrected. That is, the mean value of the 500 msec preceding the feedback was subtracted from all values of the data segment. All subsequent analyses are based on these data segments.

Due to general arousal effects, there was a large decrease in pupil size during the first few trials, which is independent from the task manipulation. To address this, data from the first five trials of the task were not analyzed. Furthermore, the task ended after the feedback for the last trial; so in this trial, no 6-second long pupil data segment could be extracted. Thus, the last trial of each run was also removed from pupil analyses. Importantly, for analyzes not involving pupil data, no such exclusions were done.

The effect of task variables (feedback type, reversal) and model estimates (state entropy, state change probability) on pupil responses were examined using a series of linear mixed models separately for each time point after the feedback. That is a separate regression model was used for each of the 350 time bins representing the 6 seconds after the presentation of the feedback. In each of these regression models, the outcome variable was the pupil size in that given time bin for each trial, whereas we entered different fixed and random effects, as predictors. First, the mean pupil size during the baseline period, which also was used for the baseline correction procedure (see above) was entered as a nuisance variable to control for the negative correlation between baseline pupil size and evoked responses (for similar approaches, see Filipowicz et al., 2020). Second, participant identity was added as a random effect. Beside these two predictors, the variable of interest for the specific analysis (trial characteristic or model estimate) also was added to the mixed models. In each case, we tested whether this variable of interest exerts a significant influence on evoked changes, by using a likelihood ratio test (LRT, for its use in linear-mixed model testing, see Brown, 2021). We compared whether the model with the variable of interest is more likely to be responsible for the variation in pupil size changes compared with a model without the effect of this variable (i.e., only with baseline pupil size as fixed, and participant as random effect).

As we conducted the same LMM analysis on all 350 bins, cluster based permutation testing was performed to control for multiple comparisons. This method determines the minimum length of subsequent time bins with significant LRT results (i.e., clusters), which is unlikely to manifest by chance. Each LMM was conducted 500 times, and the variables of interest (feedback type, reversal, state entropy, state change probability) were randomly permuted during each run. The result of the LRTs for each relevant predictors was saved for each time point and for each permutation. Then, we summarized cluster sizes of subsequent significant LRTs during all permutations and examined the distribution of these cluster size values; specifically, we computed the 95^th^ percentile of the distribution, and interpreted clusters of significant LRTs in the LMM observed data only if the cluster size exceeded this threshold.

The analyses were run in the R statistical language (R Development Core Team, 2019), using the package “lme4” (Bates et al., 2015). Other data analysis involving the comparison of real and simulated data and model estimates was done in JASP (JASP Team, 2022). The normal distribution of the underlying variables was tested using the Shapiro-Wilk test. For normally distributed variables, parametric (paired t-test, ANOVA), whereas for nonnormally distributed variables, nonparametric tests were used (e.g., Wilcoxon signed-rank test).

## Results

### Comparison of model predictions and behavioral performance

In Exp 1, switches in reward contingencies were triggered after the participant chose the preferred option in eight consecutive choices. On average, there were 4.34 (SE = 0.42 range: 1-8) switches during the 150 trials. Only 5 of the 26 participants suggested in the debriefing that their own choices had an effect on where the stone was hidden. In Exp 2, switches occurred independently from the participants’ behavior, with a probability of 0.06 in each trial. Consequently, there were on average 5.92 (SE = 0.44 range: 2-12) switches in Exp 2a and 5.78 (SE = 0.37 range: 2-12) switches in Exp 2b.

**Fig. 3:**
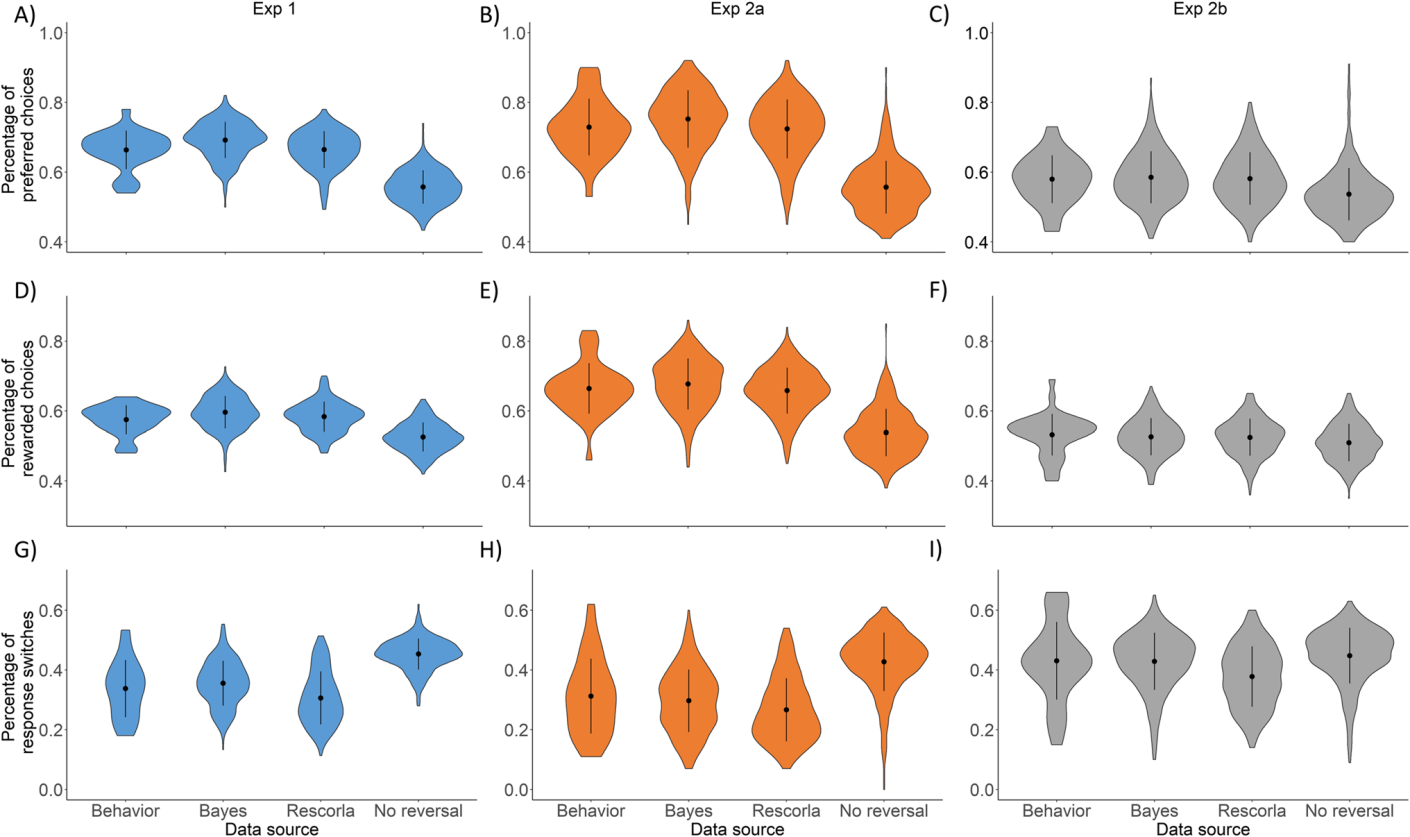
Violin plots showing summary measures of behavioral performance and simulated data based on model predictions for all experimental conditions. **A-C** Percentage of preferred choices in the three experimental conditions, calculated from the observed behavior, and the simulations of the different models. **D-F** Percentage of rewarded choices in the three experimental conditions, calculated from the observed behavior, and the simulations of the different models. **G-I** Percentage of response switches in the three experimental conditions, calculated from the observed behavior, and the simulations of the different models. Note: error bars represent standard deviation

The mean proportion of choices, where participants chose the preferred response option is shown in Fig. 3A-C (left violin pont). This proportion was above the chance level in all three conditions (all *t*s > 6.18, all *p*s < 0.001, all *d*s > 1.01 using one-sample *t*-test with test value of 0.5), suggesting that participants could figure out which choice option is more advantageous. Furthermore, using independent samples t-test, we can show that participants chose the preferred choice option more frequently in Exp 1 (0.75/0.25 condition) than in Exp 2b (0.65/0.35 condition), *t*(61) = 5.17, *p* < 0.001, *d* = 1.33, but less frequently than in in Exp 2a (0.85/0.15 condition), *t*(61) = 3.56, *p* < 0.001, *d* = 0.91. That is, the proportion of trials where participants chose the preferred response option scaled with the level of expected uncertainty in the three conditions.

As can be seen Fig. 3D-F (left violin plot), a similar pattern was observed for the proportion of rewarded choices (i.e., where participants received reward for their choices, regardless of whether they chose the preferred or the not preferred response option). Similar to the previous analysis, the percentage of rewarded choices scaled with the level of expected uncertainty (Exp 1 – 0.75/0.25 condition vs. Exp 2b – 0.65/0.35 condition: *t*(61) = 3.17, *p* = 0.002, *d* = 0.81; Exp 1 – 0.75/0.25 condition vs. Exp 2a – 0.85/0.15 condition: *t*(61) = 5.68, *p* < 0.001, *d* = 1.45) and exceeded chance level in all three experimental conditions (all ts > 3.34, all *p*s < 0.002, all *d*s > 0.55 using one-sample *t*-test with test value of 0.5).

We also calculated the percentage of trials where participants chose different response option, as in the previous trial. As can be seen in Fig. 3G-I (left violin plot), such response switches were more common in Exp 2b (0.65/0.35 condition), compared with Exp 1 (0.75-0.25 condition),* t*(61) = 3.11, *p* = 0.003, *d* = 79, whereas no difference was observed between Exp 1 (0.75-0.25 condition) and Exp 2a (0.85-0.25 condition), *t*(61) = 0.87, *p* = 0.39, *d* = 0.22.

To investigate the generative performance of the models (whether they can correctly mimic the behavior of real participants), we repeated the above analyses using model simulations. The simulations were performed with each considered model similar to the model recovery test. The tasks and task parameters were the same as in the experiments (Exp 1, Exp 2a, and Exp 2b).

Crucially, all tasks were simulated independently for each model and also independently from the experimental sessions. This is especially important in the case of Exp 1, where reversals are contingent on the participant’s actions. In the simulations, this depended on the simulated model responses. The simulated model responses are determined by first calculating the choice probabilities using the choice model and then sampling from that distribution.

We used the model parameter vectors obtained by the maximum likelihood parameter fitting of the experimental data. This time (unlike for the recovery test) we generated ten independent sessions for each fit parameter vector to reduce sampling noise. We treated these as sessions of different simulated participants.

The three behavioral measures (percentage of preferred/rewarded choices and response switches) also were calculated for the model simulations. As shown in Fig. 3, the distribution of the predicted choices by the Bayes and Rescorla model closely matched participants’ choices, whereas the no reversal model produced somewhat different pattern of result.

We also investigated how real behavior of participants and simulated behavior of the different models changed after reversals. Reversals should trigger an increase in the proportion of response switches. Furthermore, they should lead to a significant drop in the number of preferred and rewarded choices, followed by an increase in the subsequent trials, as the changes in SRCs are detected.

**Fig. 4:**
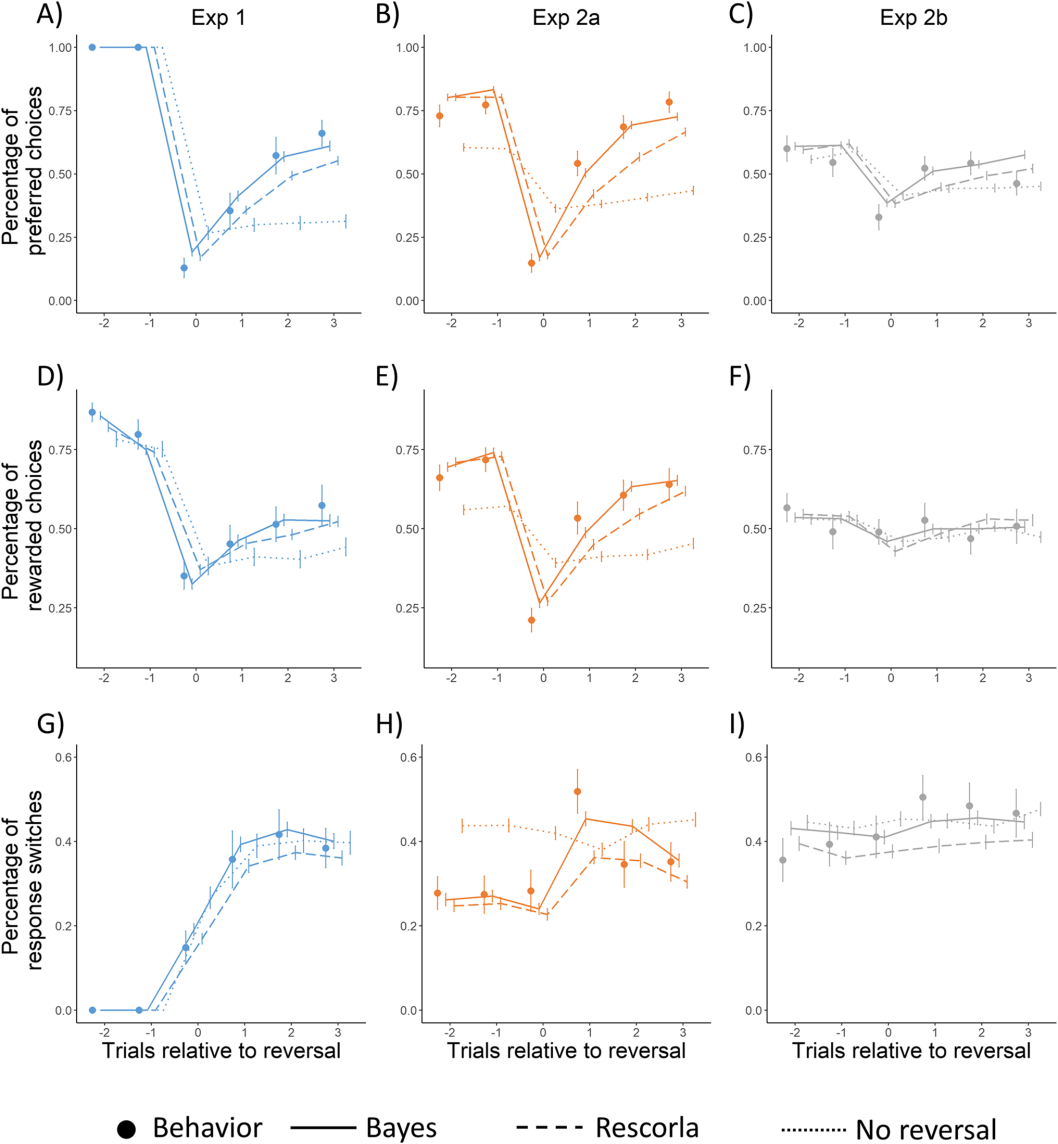
Effect of reversal on behavioral performance and simulated data based on model predictions. **A**-**C** The percentage of choosing the preferred, advantageous response option in the three experimental condition—individual points: participants’ choices, solid/dashed/dotted line: simulations with the different models. **D**-**F** The percentage of choosing the rewarded response option in the three experimental condition—individual points: participants’ choices, solid/dashed/dotted line: simulations with the different models. **G**-**I** The percentage of changing the chosen response option from the last—individual points: participants’ choices, solid/dashed/dotted line: simulations with the different models. Reward contingencies are reversed at trial 0. Note: Error bars denote the standard error of the mean

Regarding Exp 1 and Exp 2a, this pattern can be clearly observed, as shown in Fig. 4, for both observed behavior (dots in Fig. 4A-B, D-E), and for data acquired through simulation using the Bayes and the Rescorla model (solid and dashed lines, respectively in Fig. 4A-B, D-E). The drop between the last trial before the first trial after reversal, and the increase from the first to the second trial was significant for preferred and rewarded choices, for both Exp 1 and Exp 2a, and for both real and simulated data using the Bayes or the Rescorla model (tested using Wilcoxon signed-rank test, all *p*s < 0.01, all *r*s > 0.26, with the exception of the increase after reversal in the proportion of choosing the rewarded response option in Exp 1, *W* = 27, *p* = 0.21, *r* = 0.41). The effect of reversals also is evident on response switches (Fig. 4G-H). Its proportion increases significantly between the first and the second trials after the reversal, that is, when participants could process the first feedback regarding the changing SRCs (tested using Wilcoxon signed-rank test, all *p*s < 0.01, all* r*s > 0.47, for both observed behavior and simulated data with the Bayes or the Rescorla model). Note, that behavior simulated using the no reversal model failed to show the above pattern consistently (see dotted lines in Fig. 4A-B, D-E, G-H); whereas the percentage of preferred and rewarded choices dropped after the reversal significantly in both experiments (tested using Wilcoxon signed-rank test, all *p*s < 0.05, all *r*s > 0.20), no subsequent increase was evident (tested using Wilcoxon signed-rank test, all *p*s > 0.16, all* r*s > 10), and the percentage of response switches increased in Exp 1 (*W* = 1,340, *p* < 0.001, *r* = 0.44), but decreased in Exp 2a (*W*=17537, *p* = 0.03, *r* = 0.15).

In Exp 2b, the behavior of the participants and also the predictions of the model were less sensitive to reversals. Whereas the proportion of preferred and rewarded choices dropped after reversals in both real and simulated data, subsequent increase was only observed for a subset of the data sets. Preferred choices increased significantly after the reversal in the observed data set, and in the simulations of the Bayes and the Rescorla model, whereas rewarded choices increased significantly in the stimulations of the Bayes and the Rescorla model (tested using Wilcoxon signed-rank test, all *p*s < 0.02, all rs > 0.16). Furthermore, reversals did not lead to an increase in response switches in none of the data sets (tested using Wilcoxon signed-rank test, all *p*s > 0.09, all rs < 0.38). This pattern of results indicates that such high level of noise complicates the separation of random noise and systematic changes in SRCs.

Importantly, examination of Fig. 4 indicates that the changes triggered by reversals are largely similar for observed behavior and simulated data using the Bayes and the Rescorla model. In contrast, data acquired through simulation with the no reversal model failed to show a similar pattern. This further supports the results of the model validation (Fig. 2C), which suggested significantly worse fit for the no reversal model than the other two models. Furthermore, we can also observe that behavior simulated using the Bayes model, as compared to the Rescorla model, produces quicker learning after the reversal, and it also better approximates the behavior of the real participants. To test this observation, we calculated mean values of preferred choice and response switch percentage values for the second to the fourth trials after the reversal (shown as trials 1-3 in Fig. 4) and compared the Bayes and the Rescorla model performance: the behavior simulated using the Bayes model led to larger preferred choice and response switch percentage in all three experiments (tested using Mann-Whitney *U* test, all *p*s > 0.001, all* r*s > 0.14). This also supports the results of the model validation procedure, which suggested a better fit for the Bayes, as compared to the Rescorla model.

### Link between trial characteristics and model estimates of unexpected uncertainty under different levels of expected uncertainty

As described in the *Introduction*, we aimed to separate two aspects of unexpected uncertainty: probability of change in SRCs, and the uncertainty evoked by the invalidity of previously established SRCs. These two aspects were quantified by (1) the probability that the preferred response option has changed (state change probability), and (2) the entropy of participants’ beliefs about the preferred location (state entropy). We investigated the properties of these two quantities on model estimates calculated for the feedback sequences recorded during the experimental sessions. We used model parameters fitted to the actual participant’s responses using the maximum likelihood method.

**Fig. 5:**
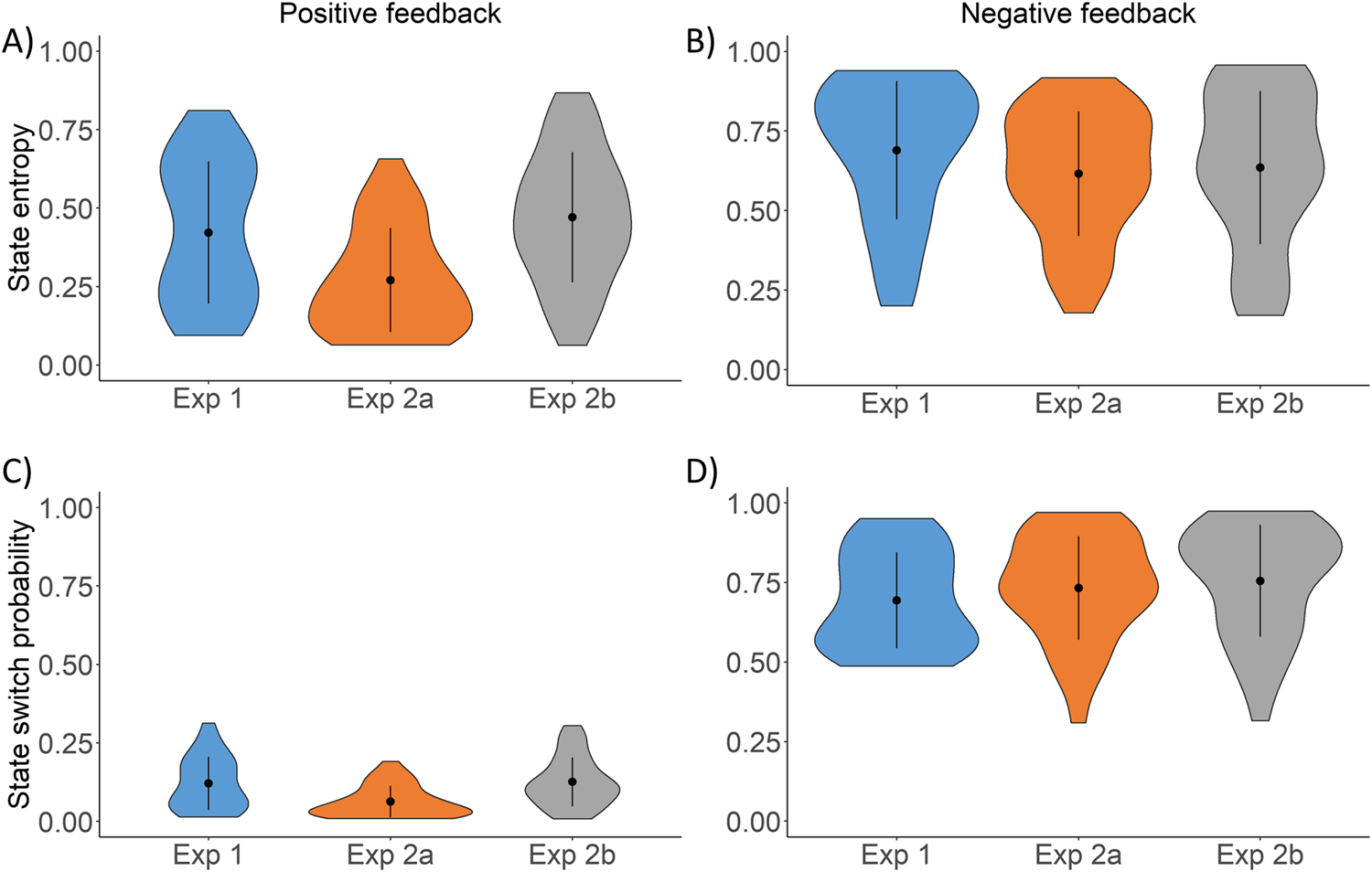
Violin plots for the model estimates after different feedback types. **A-B** State entropy after positive (**A**) and negative (**B**) feedback in the three experimental condition. **C-D)** State change probability after positive (**C**) and negative (**D**) feedback in the three experimental condition. Note: error bars represent standard deviation

If we assume that participants follow task instructions and always chose the response option that they think is the preferred one, then negative feedback signalizes either that the SRCs have changed or that in this trial the nonpreferred response option was rewarded. Although the participant cannot decide between these two possibilities based on a single trial, negative feedback always increases the probability that the preferred option has been changed and in general should lead to more uncertainty regarding the validity of the currently established SRCs. Therefore, we expected higher state entropy and state change probability estimates after negative as compared to positive feedbacks. This was indeed the case, as can be seen Fig. 5, when comparing Fig. 5A versus 5B and Fig. 5C versus 5D (the difference between positive and negative feedback is significant for both measures, and for all three experimental conditions, tested using Wilcoxon signed-rank test, all *p*s < 0.001, all *r*s = 1).

**Fig. 6:**
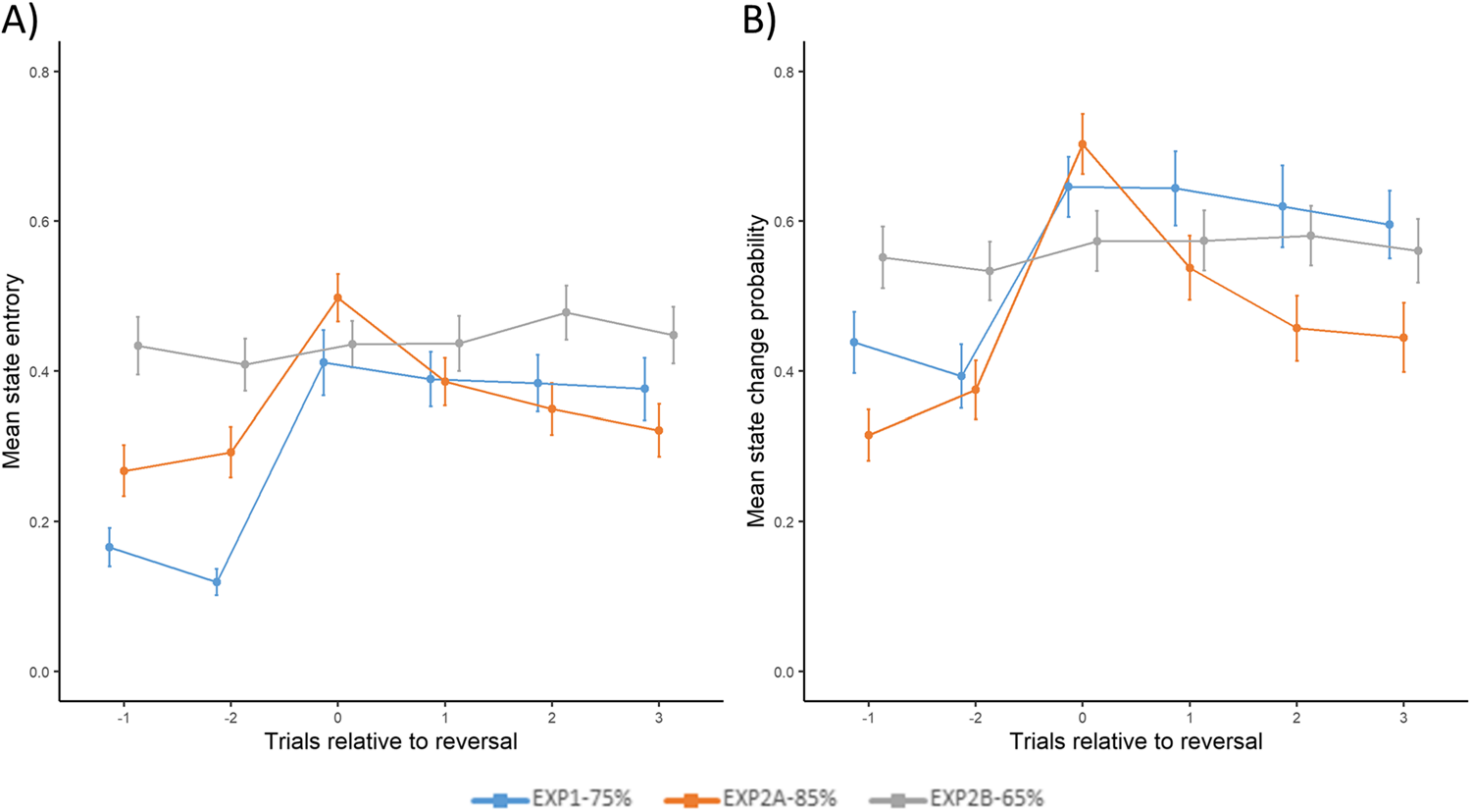
Effect of reversal on model estimates. **A** State entropy. **B** State change probability. Reward contingencies are reversed at trial 0. Error bars denote the standard error of the mean

We also investigated how state entropy and state change probability are affected by reversals. As shown in Fig. 6A-B, in line with both real behavior and simulated data shown in Fig. 4, model estimates are differently affected by reversals in the three conditions. For the 0.65/0.35 condition, reversal did not affect state entropy and state change probability significantly, whereas in the two other conditions, significant increase in both estimates was triggered after the first feedback was received following the reversal (testing the difference between the trial before and after the reversal using Wilcoxon signed-rank test: Exp 1, state entropy: *W* = 14, *p* < 0.001,* r* = 0.89; state change probability: *W* = 9, *p* < 0.001, *r* = 0.94; Exp 2a, state entropy: *W* = 51, *p* < 0.001, *r* = 0.82; state change probability: *W* = 8, *p* < 0.001,* r* = 0.96; Exp 2b, state entropy: *W* = 275, *p* = 0.26, *r* = 0.22, state change probability: *W* = 281, *p* = 0.78, *r* = 0.05). Importantly, in Exp 1, reversals occurred after eight consecutive trials where participants choose the preferred response option. Such behavior indicates strong beliefs about the preferred option. The fact that reversals in this condition are accompanied by a steep increase in state entropy further validates that state entropy can be used to measure unexpected uncertainty.

### Link between trial characteristics and feedback-evoked pupil size changes under different levels of expected uncertainty

**Fig. 7:**
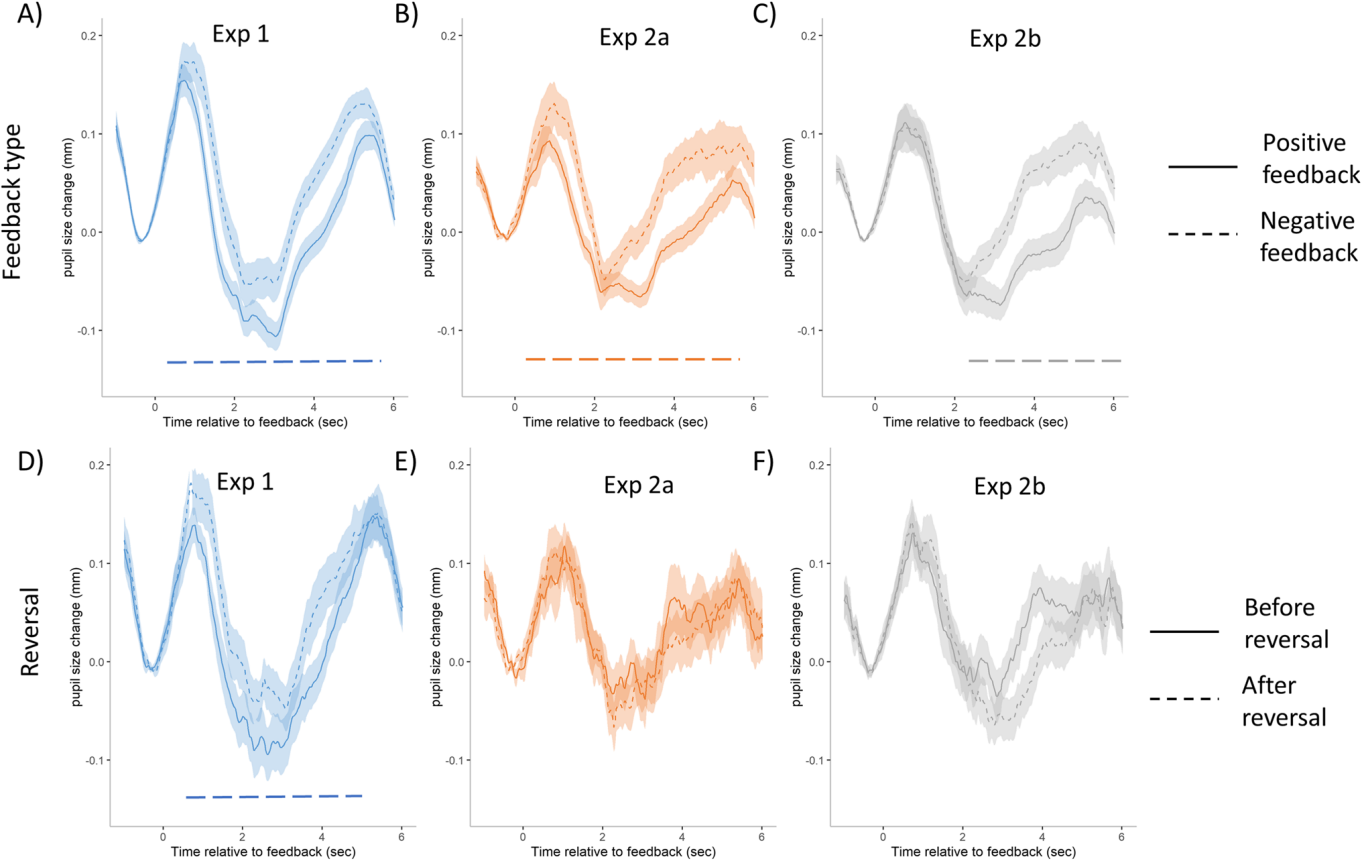
Feedback evoked pupil size changes, as a function of feedback type and reversal. **A-C** Feedback evoked pupil size after positive (solid line) and negative (dashed line) feedback in the three experimental conditions. **D-F** The difference between the two trials preceding (solid line) and following (dashed line) reversals in the three experimental conditions. Pupil size values are aligned to the start of the feedback, and all trials are baseline corrected by subtracting the mean pupil size of the 500 msec before the feedback from all data points of the trial. Shading represents the between-subject standard error of the mean. Dashed horizontal lines at the bottom of the figures signalize a significant likelihood ratio test of feedback type or reversal in the given time bin

As a next step, we investigated how feedback evoked pupil responses are related to trial characteristics (reversals and positive/negative feedback), under different levels of expected uncertainty. Figure 7A-C shows feedback-related pupil size changes for the three experimental conditions, separately for trials with positive and negative feedback. As can be seen, both types of feedback evokes a marked phasic pupil response. The significance of the differences was tested using a series of linear mixed models, separately for each time point after the feedback and testing the effect of feedback type (see *Method* for details). The dashed horizontal line in Fig. 7A-C indicates time periods where the likelihood ratio test is significant. Negative feedback leads to larger pupil size for all three experimental conditions, but for Exp 2b (0.65/0.35 condition), this difference is only significant for the late period in the trial (starting from 2 seconds after the feedback). Thus, we can conclude that pupil-linked brain arousal was sensitive to negative feedback, with negative feedback triggering larger evoked pupil response.

In a similar set of analyses, we also investigated the effect of reversals on evoked pupil size changes. In this analysis, we entered data from trials, which directly preceded or followed a reversal in SRCs. Specifically, the last two trials before and the first two trials after reversals were entered. Figure 7D-F shows the pupil responses for trials preceding and following reversals, respectively. Similar to the effects of feedback, the effects of reversals also were investigated by using a series of linear mixed models. Horizontal dashed lines in Fig. 7D-F shows the results of the linear-mixed models investigating the effects of reversal—as shown, trials after a reversal were associated with increased feedback-evoked pupil response in Exp 1, whereas no such difference was evident for Exp 2a or Exp 2b. Thus, reversals were associated with increased feedback-evoked pupil responses only after Exp 1.

### Effect of state entropy and state change probability in predicting feedback-evoked pupil dilation

**Fig. 8:**
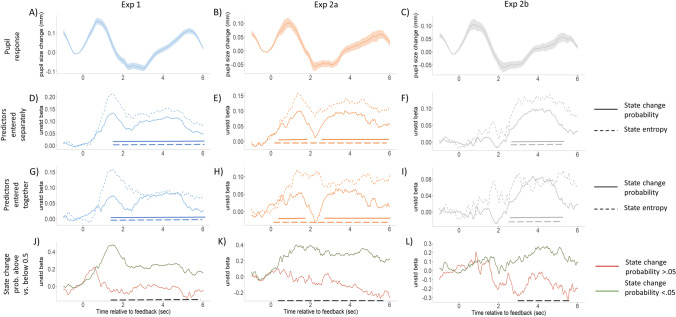
Effect of state entropy and state change probability on feedback-evoked pupil size changes. **A-C** Mean pupil responses in the three experimental conditions. **D-F** Unstandardized regression coefficient of state change probability (solid line) and state entropy (dashed line), regressed on baseline corrected pupil size in the different time bins. The model estimates are entered in separate regression models. Horizontal lines at the bottom of the figures signalize a significant likelihood ratio test of state change probability (solid line) or state entropy (dashed line) in the given time bin. **G-I** Unstandardized regression coefficient of state change probability (solid line) and state entropy (dashed line), regressed on pupil size in the different time bins. The model estimates are entered in the same regression model. Horizontal lines at the bottom of the figures signalize a significant likelihood ratio test of state change probability (solid line) or state entropy (dashed line) in the given time bin. **J-L** Unstandardized regression coefficient of state change probability regressed on pupil size in the different time bins, separately for trials with state change probability below 0.5 (green line) and above 0.5 (red line). Horizontal lines at the bottom of the figures signalize a significant likelihood ratio test for the interaction between state change probability and trial type (state change probability below/above 0.5)

Finally, we investigated how state entropy and state change probability affect evoked pupil-size changes. Similar to the previous section, we constructed a linear mixed model for each time point, with the pupil size as outcome variable, participant as categorical random variable, and baseline pupil size, state change probability and/or state entropy as fixed effects. In the first set of analyses, we only entered one of the two model estimates (either state entropy or state change probability) into the model, whereas in the second set of analyses, the two predictors were entered together. The results of these analyses are shown in Fig. 8, with the mean pupil response presented for each experimental condition in Fig. 8A-C, whereas the results of the different regression models are shown in Fig. 8D-L.

When examining its effects separately, we can observe that both state entropy and state change probability exerts a significant linear effect on evoked pupil size changes. Figure 8D-F shows unstandardized regression coefficients for both state change probability (solid lines) and state entropy (dashed lines), as sole predictors, over the course of the post-feedback period. The significance of these predictors was tested using likelihood ratio test, and solid and dashed horizontal lines at the bottom of each figure indicate at which time bins is the effect of the corresponding predictor significant. In the case of Exp 1 and Exp 2a, the effects of both state change probability and state entropy are positive and significant for the whole duration of the post-feedback period. In contrast, and similar to the effects of feedback type, in Exp 2b the effects of the model estimates are only significant for the late period in the trial (starting from 2 seconds after the feedback).

In a second set of analyses, we examined a model to determine pupil size changes in which both state entropy and state change probability are present as linear predictors. It is important to note that state entropy is deterministically related to state change probability, forming an inverted U-shaped relationship: it is maximal when the state change probability is 0.5, and minimal, when it is either 0 or 1. Due to this deterministic relationship, the regression model involving both predictors can be interpreted as a linear mixed model with basis functions. The state change probability predictor has two basis functions: the Φ_0_(*x*) = *x* linear/identity and the Φ_1_(*x*) = *x* ∙  *log* (*x*) + (1 − *x*) ∙  *log* (1 − *x*) entropy functions. (Note that this analysis is analogous to a polynomial regression with Φ_0_(*x*) = *x* linear and Φ_1_(*x*) = *x*^2^ quadratic terms, only with different basis functions applied.) The results of this analysis are presented in Fig. 8G-I. Similar to the previous sections, nonstandardized regression coefficients of both state change probability (solid lines) and state entropy (dashed lines) are shown. Horizontal lines again signalize the significance of the likelihood ratio test, conducted in a similar way than in the previous analysis. As the figures show, both state change probability and state entropy exerted a significant linear effect on pupil size changes in all experimental conditions even if the linear effect of the other variable has been taken into account. The effects were constrained to the late part of the post-feedback period for Exp 2b.

These findings should be interpreted with respect to the basis functions applied. The positive and significant coefficients observed for the linear term (i.e., state change probability) shows that with increasing probability of change in the SRCs, the evoked pupil response tends to increase—that is, the mean evoked pupil response is higher for high compared to low state change probability values. The positive and significant coefficient observed for the second, entropy term indicates that there is a concave curvilinear relationship between state change probability and evoked pupil size. This suggests that medium state change probability values may lead to larger pupil dilation compared with low or high values.

To further interpret the curvilinear relationship represented by the significant state entropy term, one has to consider the fact that state change probability and state entropy are positively correlated when the former is smaller than 0.5, whereas they are negatively related when values of state change probability are above 0.5. Thus, when the probability of change is below 0.5, then an increase in state change probability is associated with an increase in uncertainty (i.e., it becomes less and less certain that the preferred side is still unchanged), whereas for change probabilities above 0.5, increase in state change probability is associated with a decrease in uncertainty (i.e., it becomes more and more certain that a switch has occurred and now the opposite side is preferred). That is, the positive linear effect of state entropy suggests that state change probability might be related differently to pupil size changes for values below and above 0.5. This can be confirmed by examining the effect of state change probability separately for trials with state change probability values below and above 0.5 (low change probability vs. high change probability trials). The effect of state change probability for these two types of trials separately is shown on Fig. 8J-L (with green line showing the effect for low change probability trials, whereas a red line showing the effect for high change probability trials). As shown on these figures, the effect of state change probability on evoked pupil size changes differs for low versus high change probability trials. The significance of this interaction effect was tested by using a linear mixed model, which included a dichotomous variable coding whether state change probability is higher or lower than 0.5, and investigated whether the interaction of this variable with state change probability is significant or not. The black dotted line at the bottom of Fig. 8J-L signalizes that there is indeed a significant interaction.

To sum up, both state change probability and state entropy significantly and positively predicted evoked pupil size changes in all experimental conditions. Our result suggests that the increase in state change probability has a significant linear effect on evoked pupil responses, that is irrespective the uncertainty, larger state change probability is associated with larger evoked responses. This relationship, however, is altered by the fact, that state entropy, which is the highest for medium state change probability values also has a significant positive linear effect on pupil size even after the linear effect of state change probability is taken into account.

## Discussion

In two experiments, we investigated how the level of expected and unexpected uncertainty affects choice behavior, task-related beliefs, and pupil-linked brain arousal. We used a probabilistic reversal learning task, where participants had to figure out which of two response options is linked to reward with higher probability—importantly, this advantageous response option was switched repeatedly during the task.

The level of expected uncertainty was manipulated by varying the reward probability for the advantageous, preferred response option in different experimental conditions (Exp 1: 0.75, Exp 2a: 0.85, Exp 2b: 0.65) and symmetrically also for the disadvantageous, non-preferred response option (Exp 1: 0.25, Exp 2a: 0.15, Exp 2b: 0.35). Our results indicate that varying SRCs in such a way influences choice behavior and the detection of reversals and also might influence how feedback-evoked pupil responses are linked to feedback type and reversals, in particular for high levels of expected uncertainty.

To assess the two aspects of unexpected uncertainty (change probability and the resulting uncertainty), we used a Bayesian model to estimate participants’ beliefs about which of the two response options might be the advantageous response option. In line with previous research (De Berker et al., 2016; Filipowicz et al., 2020; Krishnamurthy et al., 2017; Lavín et al., 2014; Nassar et al., 2012; O’Reilly et al., 2013; Preuschoff et al., 2011; Van Slooten et al., 2017; Zénon, 2019), we found that pupil-linked brain arousal was sensitive to the level of unexpected uncertainty. In addition to these results, we showed that unexpected uncertainty affects pupil responses in two independent routes: both high uncertainty regarding the valid SRCs and also high change probability of valid SRCs were associated with larger feedback evoked pupil responses.

### Disentangling the effects of expected and unexpected uncertainty

Our main manipulation was to vary the level of expected uncertainty in the three conditions of the two experiments. This clearly affected performance, as participants’ ability to correctly choose the preferred choice option declined with increasing level of expected uncertainty. This pattern might be caused by the fact that high levels of expected uncertainty hinder the identification of reversals. With increasing level of expected uncertainty, it becomes increasingly difficult for participants to differentiate whether reward omissions are due to random variation inherent in the established SRCs (i.e., expected uncertainty) or due to reversals (i.e., unexpected uncertainty). In line with this, the level of expected uncertainty moderated how reversals affected behavior and model estimates: in Exp 2a (low expected uncertainty), participants quickly figured out the change in SRCs, as evidenced by the fact that there was only a temporary change in the proportion of preferred choices and response switches. Similarly, both model estimates increased for only one trial and decreased thereafter. In contrast, in Exp 1 (medium expected uncertainty), the reversal had a more enduring effect on both behavior and model estimates. Finally, in Exp 2b (high expected uncertainty), reversals exerted no noticeable influence on behavioral measures or model estimates. This pattern can be explained by the fact that reward omissions after reversals might have signaled expected and unexpected uncertainty with different probabilities. In the 0.65/0.35 condition (Exp 2b), due to the high level of expected uncertainty, negative feedbacks can be attributed to random noise, and so the level of unexpected uncertainty does not increase much after reversals, and thus behavior is not adjusted accordingly. In the 0.85/0.15 condition (Exp 2a), due to low level of expected uncertainty, negative feedbacks signal reversal with high probability, so new reward probabilities can be quickly established; thus behavior is adjusted quickly and unexpected uncertainty falls back rapidly. Finally, in the 0.75/0.25 condition (Exp 1), negative feedback might be attributed both to random noise and to reversal, so additional sampling of evidence is required before new reward probabilities can be formed; thus, the level of unexpected uncertainty remains large for several trials and behavior is adjusted only gradually.

We also examined how different levels of expected uncertainty affect pupillary responses observed during the processing of the feedback. First, we investigated how the type of feedback affects feedback-evoked pupil responses and found that negative feedback was associated with larger evoked-pupil response. This effect aligns well with the fact that negative feedback is associated with larger state entropy and state change probability estimates. That is, irrespective of the level of expected uncertainty, negative feedback is treated as a sign that the SRCs might have changed. Interestingly, in Exp 2b, the pupil response evoked directly by feedback processing (i.e., 0-2 sec after the feedback) was of similar magnitude for both positive and negative feedbacks, and the difference between the responses emerged only in the late period (see below for possible explanation for this effect). This pattern of result is a replication of the findings reported by Nassar et al. (2012), who showed that prediction errors lead to larger evoked pupil size changes in the time period of 0-2 sec after the feedback, if the level of expected uncertainty is low compared with the case when it is high. In our case, prediction error can be conceptualized as a negative feedback, and our results show that prediction errors only led to larger pupil size change if the level of expected uncertainty was low or medium (i.e., Exp 1 and Exp 2a), but not when it was high (i.e., Exp 2b).

We also investigated whether the level of expected uncertainty modulates how pupil-linked brain arousal is affected by reversals (i.e., the “objective” trigger of unexpected uncertainty). To this goal, we compared feedback-evoked pupil responses for the two trials preceding and following the reversal, respectively, and found that pupil responses were larger after the reversal in Exp 1 (moderate expected uncertainty) but not in Exp 2a or Exp 2b (low or high levels of expected uncertainty). Differences in task design might explain this pattern. In Exp 1, reversals were triggered only when participants chose the preferred response option eight consecutive times, which might have led to an efficient detection of reversals, whereas in Exp 2, the occurrence of reversals was random, so they might have occurred also on trials in which the participant were still uncertain about the preferred response option. Furthermore, the low number of reversals also might have led to low statistical power to show differences in Exp 2a and Exp 2b. Thus, further research controlling for the above factors is required to examine whether the level of expected uncertainty affects pupil responses after reversals.

Regarding model estimates of unexpected uncertainty, our results suggest that both change detection and resulting uncertainty predicts pupil-linked brain arousal, as both model estimates (state entropy and state change probability) were positively linked to feedback-evoked pupil responses in all experimental conditions. This pattern of results suggests that both aspects of unexpected uncertainty are involved in pupil-linked brain arousal. Furthermore, our design of only two response options and deterministically linked model estimates for change probability and resulting uncertainty enabled us to show that they exert an independent influence on pupil responses; pupil responses were larger when the change probability was high compared with the cases when it was low. Simultaneously, the large uncertainty regarding the SRCs by medium levels of change probability was positively linked to pupil responses, as evidenced by the demonstrated curvilinear relationship.

Importantly, for low and medium levels of expected uncertainty (Exp 1 and Exp 2a), the effects of unexpected uncertainty and feedback type were present during the whole post-feedback period. In contrast, they only appeared two seconds after the feedback presentation in Exp 2b. This pattern might be related to the fact that the pupil-size changes directly evoked by the feedback reflect a more automatic processing related to reinforcement learning, whereas later stages might involve effortful decision processes associated with the next choice. The absence of the early effects of both unexpected uncertainty and feedback type in Exp 2b suggests that at high levels of expected uncertainty, the differentiation between changes in SRCs and the noise inherent in the SRCs is difficult. This is reflected then in the low behavioral performance and also in the insensitivity of pupil-linked brain arousal to unexpected uncertainty directly after the presentation of the feedback.

Finally, our results complement a recent study by Filipowicz et al. (2020), who used a similar probabilistic reversal learning task and investigated how varying reversal probability by constant expected uncertainty (SRC: 0.80/0.20) affects pupil-linked brain arousal. They showed that pupil-linked brain arousal was sensitive to surprise and low strength of the belief about the location of the reward. In contrast to their study, we varied SRCs and held reversal probability constant (at least for the two conditions of Exp 2) but still observed similar correlates of pupil-linked arousal. Thus, the combined results of these two studies suggest that pupil-linked brain arousal is sensitive to unexpected uncertainty in very different contexts of probabilistic reversal learning.

### Neuromodulation, expected, and unexpected uncertainty

It was suggested that pupil size changes are correlated with neural activity in the locus coeruleus (LC), a brainstem nucleus from which noradrenergic cortical transmission originates (Aston-Jones & Cohen, 2005; Joshi et al., 2016; Joshi & Gold, 2020; Murphy et al., 2014). Because of this, our results are relevant in assessing theories and models discussing the role of the LC/NA system in behavioral regulation and information processing.

Most relevantly, the computational theory put forward by Yu and Dayan (2002, 2005) suggests that changes in unexpected uncertainty are linked to changes in noradrenergic activity, whereas expected uncertainty is coded in the brain by means of cholinergic neurotransmission. Our results support this differentiation by showing that unexpected uncertainty leads to sustained increase in pupil size (i.e., differences in the late time period after the feedback), whereas they also suggest that the two concepts of uncertainty, and possibly the activity of the underlying neurotransmitter systems, are not independent of each other: high levels of expected uncertainty (noise) hinders the detection of “true” changes in the environment (see also limitations regarding the conceptualization of the two concepts).

Other theories focus on the role of the LC/NA system in encoding links between stimuli, responses, and outcomes. The adaptive gain theory suggests that LC based NA modulation codes the availability and utility of currently available rewards, and by this governs the shift between exploitation of current rewards and exploration of unknown, potentially larger rewards (Aston-Jones & Cohen, 2005). During exploration, LC neurons are characterized by high tonic firing rates and an absence of spike activity (tonic mode), whereas during exploitation, lower tonic firing rates and task-related spike activity can be observed (phasic mode). Recently, Sales et al. (2019) put forward a computational model building on the idea of active inference (Friston et al., 2017), which suggested that this dual-mode of LC-activity can be explained by the sensitivity of the LC to the discrepancy between the predicted and observed probability of different world states and action outcomes (state-action prediction errors, SAPE). According to the model, stimuli not predicted by the current world model always generate SAPE and so lead to transient increase in LC firing level (phasic LC responses). Importantly, when there are drastic and sustained changes in underlying contingencies messing up model predictions, then sustained SAPE will be present and a tonic firing of the LC neurons will be triggered leading to model update. Finally and in accordance with these models, several authors suggested that noradrenergic modulation influences the learning rate, i.e., to which degree new, nonpredicted observations can alter the existing world model (Jepma et al., 2016; Krishnamurthy et al., 2017; Nassar et al., 2012).

Our results support the above models, as unexpected uncertainty discredits previously correct SRCs and so might trigger explorative behavior and increase the rate of learning to settle new SRCs and update the world model. In line with this, pupil size changes triggered by unexpected uncertainty in our experiments lead not only to transient increase during processing the feedback (i.e., the first 2 seconds of the post-feedback period) but were also followed by more enduring changes, which led to an increase in pupil size before the next trial (i.e., 3-6 seconds in the post-feedback period). Thus, our results add further evidence to earlier data suggesting that pupil-linked brain arousal signals changes in the exploration/exploitation trade off (Murphy et al., 2014; Pajkossy et al., 2017, 2018) and also provide support for suggestions linking pupil-linked brain arousal specifically to model-updating (Sales et al., 2019; Zenon, 2019) and learning rate-adjustment (Jepma et al., 2016; Krishnamurthy et al., 2017; Nassar et al., 2012).

## Limitations

In a recent study, it was shown that not only LC-related NA, but also midbrain-related Ach, activity is reflected in pupil size changes (Reimer et al., 2016). If this link also is present in humans, then due to the suggested link between ACh and expected uncertainty (Yu & Dayan, 2002, 2005), a direct link between pupil size changes and expected uncertainty could be also shown (as reported by De Berker et al., 2016). It must be noted, however, that in our paradigms the effects of expected and unexpected uncertainty were not independent of each other, as we were interested in how decision makers disentangle their respective influence. Thus, our design might be suboptimal to investigate how expected uncertainty, per se, affects pupil size. It might be the case, for example, that ACh related activity signaling the amount of expected uncertainty evolves slowly during establishing the SRCs (Bland & Schaefer, 2012). In our experiments, this process might be disturbed by the volatility introduced in our task, and so the link between ACh activity, expected uncertainty and pupil-linked-arousal remained hidden. Furthermore, we must note that due to our design, we only compared a condition with very high (Exp2b) versus a condition with very low (Exp2a) levels of expected uncertainty. It might be possible that manipulating expected uncertainty on a more fine-graded scale would have shed light on a link between pupil-linked brain arousal and expected uncertainty. To investigate this issue, further research is needed which investigates pupil-linked brain arousal in tasks with different levels of expected uncertainty and without volatility.

## Conclusions

When the outcomes of our actions do not meet our expectations, then we have to decide whether the negative feedback signalizes that our expectations are no longer valid (unexpected uncertainty) or it is only a consequence of the fact that our expectations are of probabilistic nature (i.e., expected uncertainty). We showed that this critical feature of decision making is associated with pupil-linked brain arousal: it tracks instances when the links between stimuli and rewards are changing suddenly and in a nonpredicted manner (i.e., unexpected uncertainty) and is sensitive to both sudden increases in change probability and to the resulting uncertainty. Furthermore, low probabilistic strength of these links (i.e., high expected uncertainty) impedes this function, as prediction errors due to random noise and systematic change of the environment are hard to disentangle. This result is in line with current computational proposals linking pupil-linked brain arousal and the underlying activity of the LC/NA system to signal and handle situations when previously established contingencies are no longer valid and the mental model of the world must be updated (Sales et al., 2019; Zenon, 2019).
